# Anthocyanin extract from *Lycium ruthenicum* enhanced production of biomass and polysaccharides during submerged fermentation of *Agaricus bitorquis* (Quél.) Sacc. Chaidam

**DOI:** 10.1007/s00449-021-02605-8

**Published:** 2021-07-23

**Authors:** Shan Wu, Hong-Yun Lu, Qi-He Chen, Hui-Chun Xie, Ying-Chun Jiao

**Affiliations:** 1 College of Agriculture and Animal Husbandry, Qinghai University, No.251 Ningda Road, Xining, 810016 Qinghai China; 2 Department of Food Science and Nutrition, Zhejiang University, 154 Yugu Road, Hangzhou, 310058 Zhejiang China; 3 Qinghai Provincial Key Laboratory of Medicinal Plant and Animal Resources of Qinghai-Tibet Plateau, School of Life Sciences, Qinghai Normal University, Xining, 810008 Qinghai China

**Keywords:** *Agaricus bitorquis* (Quél.) Sacc. Chaidam, Anthocyanin, Mycelia, Polysaccharide

## Abstract

*Agaricus bitorquis* (Quél.) Sacc. Chaidam (ABSC) is a wild edible fungus uniquely found in the Tibet Plateau. ABSC is rich in polysaccharides that are considered biologically active. This study aimed to determine the feasibility of enhancing exopolysaccharide (EPS) production by ABSC in shake flask culture by supplementing the fermentation medium with anthocyanin extract. Different concentrations of *Lycium ruthenicum* Murr. (LRM) anthocyanin crude extract were tested on ABSC fermentation. The activity of phosphoglucose isomerase (PGI), phosphoglucose mutase (PGM), and phosphomannose isomerase (PMI), enzymes presumably involved in EPS synthesis by ABSC, was determined. ABSC transcriptomic profile in response to the presence of anthocyanins during fermentation was also investigated. LRM anthocyanin crude extract (0.06 mg/mL) was most effective in increasing EPS content and mycelial biomass (by 208.10% and 105.30%, respectively, *P* < 0.01). The activity of PGI, PGM, and PMI was increased in a medium where LRM anthocyanin extract and its main components (proanthocyanidins and petunia anthocyanin) were added. RNA-Seq analysis showed that 349 genes of ABSC were differentially expressed during fermentation in the medium containing anthocyanin extract of LRM; 93 genes were up-regulated and 256 genes down-regulated. From gene ontology enrichment analysis, differentially expressed genes were mostly assigned to carbohydrate metabolism and signal transduction categories. Collectively, LRM anthocyanins extract positively affected EPS production and mycelial biomass during ABSC fermentation. Our study provides a novel strategy for improving EPS production and mycelial growth during ABSC liquid submerged fermentation.

## Introduction

*Agaricus bitorquis* (Quél.) Sacc. Chaidam (ABSC), commonly known as sand mushroom, is a wild underground edible fungus naturally occurring in the Qaidam Basin, Qinghai Province, China. ABSC usually presents hypertrophied fruiting bodies, low fruiting temperature, and are resistant to a hypoxic environment. ABSC is rich in proteins, minerals, and biologically active compounds, which include polysaccharides, phenolic acids, and terpenes. Extracellular polysaccharides (EPS) of ABSC have demonstrated anti-hypoxia and anti-fatigue properties [1–3], thus making ABSC valuable for potential application in disease prevention and foods. Furthermore, EPS from mushrooms have other health-promoting effects, such as anti-tumor, antiviral, antioxidant, immune regulation, hypoglycemic, and lipid-lowering [4–6]. However, current techniques used for extracting EPS from fungi commonly result in poor yield, and further use of other bioactive compounds is also limited.

Submerged fermentation has been widely applied in the cultivation of edible fungi and secondary metabolite accumulation. Optimization of fungi fermentation conditions failed to address the needs of product development and scientific research in terms of promoting mycelia and polysaccharide production by edible fungi. The incorporation of specific exogenous substances into fungal fermentation media is known to alter cell permeability, improve metabolic processes, and stimulate fungal growth and metabolite accumulation. Specifically, the addition of vegetable oil, surfactants, and organic solvents to edible fungi liquid culture can significantly promote mycelium growth and increase the recovery of polysaccharides [7–9]. Interestingly, the addition of antioxidants was shown to effectively promote the accumulation of fungi fermentation byproducts [8].

*Lycium ruthenicum* Murr. (LRM), belonging to the Solanaceae family, is a wild spiny berry with high-stress resistance that occurs naturally in the Qinghai Tibet plateau, China. LRM contains a variety of biologically active substances [10] due to its intrinsic high levels of anthocyanins, a potent antioxidant [11, 12]. Anthocyanins serve as donors of hydrogen atoms to free radicals, thus disrupting the self-propagation chain reaction [13]. Previous studies have reported that LRM is one of the plant resources with high proanthocyanidin content, with about 5% oligomeric proanthocyanidins [14]. In addition, the variety of anthocyanins exited in LRM, of which over 80% are acylated showed strong stability [11, 15]. Currently, there are 13 species of anthocyanins in LRM [16], 10 of which have been identified [11]. Meanwhile, the anthocyanin types and components of LRM from different regions were also different [17], in which petunidin-3-glucose content in Delingha and Turin regions was the highest, about 1263.23 ± 15.97 ug/g, accounting for 97% of the total anthocyanin [18]. Anthocyanins are safe, non-toxic, and can be used in both food and medicine. Previous studies have shown that [19] anthocyanins improve lipid and glucose metabolism, and enhance antioxidant and anti-inflammatory activities and intestinal microecology. Furthermore, LRM-derived anthocyanins possess remarkable oxidation resistance, as well as anti-inflammatory and anti-aging properties, is also used as colorants. Therefore, crude extraction of anthocyanins from LRM holds promising potential for application in the healthcare, food and beverage, and pharmaceutical industries [15, 20, 21]. However, to the best of our knowledge, no prior studies have attempted to use LRM anthocyanin extract in macrofungal fermentation.

Therefore, the aim of this study was to evaluate the effect of the addition of LRM-derived anthocyanins on fermentation parameters of ABSC in a liquid medium. ABSC fermentation dynamics were determined, and the content of enzymes involved in polysaccharide synthesis was measured. The transcriptomic response profile of ABSC was investigated before and after the addition of LRM-derived anthocyanins. To enhance the synthesis of ABSC metabolites, the impact of LRM-derived anthocyanin addition on mycelium biomass and EPS content was also analyzed. Collectively, the findings presented herein serve as the basis for the development of functional foods.

## Materials and methods

### Microorganisms and culture conditions

ABSC was preserved in the Food Engineering Laboratory of Qinghai University, China. Three to five pieces (2–3 cm^2^) of activated beveled mother seed were transferred to a 150-mL triangular flask containing 80 ~ 100 mL of liquid culture medium at 26 °C in a shaking incubator at 125 rpm. Culture medium composition was as follows (g/L): maltose, 20; peptone, 5.5; MgSO_4_, 0.12; CaCl_2_, 0.1; vitamin B complex, 1.5; initial pH 6.8–7.0. Seed solution was transferred into the fermentation medium at 8% inoculation size and shaken at 125 rpm and incubated for 6 days at 26 °C. Fermentation medium composition was as follows (g/L): peeled potato, 250; maltose, 20; peptone, 5.5; MgSO_4_, 0.12; CaCl_2_, 0.1; vitamin B complex, 1.5; initial pH 6.8–7.0. Fermentation was carried out in 250-mL Erlenmeyer flasks containing 80 mL of fermentation medium and 5% inoculum in an orbital shaker incubator at 125 rpm and 26 °C for 4 ~ 5 days.

### Preparation of crude extracts of natural antioxidants from LRM

Extraction of LRM anthocyanins was performed as follows: smashed LRM was mixed with 80% ethanol solution (20:1 v/w) and placed in an ultrasonic device at 48 °C for 25 min. The residue also underwent the same extraction procedure detailed above. Anthocyanin extracts were concentrated by low-temperature vacuum distillation to eliminate ethanol, followed by vacuum freeze-drying.

### Determination of ABSC biomass and EPS content

After fermentation, ABSC mycelium was filtrated, washed three times, and freeze-dried until constant weight was obtained, which corresponded to biomass. EPS content was determined by the phenol sulfuric acid method [22]. The calibration curve of EPS was generated by linear regression of absorbance values (OD_490_) of glucose solution at different concentrations. The calibration curve showed good linearity between absorbance values (y) against reduced sugar (x; µg/mL) over the calibration range (*y* = 15.042*x* + 0.1112, *R*^2^ = 0.9982).

### Measurement of key enzymatic activities involved in EPS metabolism in ABSC

After fermentation, 0.1 g ABSC mycelium was placed in a centrifuge tube, washed three times with 20 mM phosphate buffer (pH 6.5), ground in a pre-cooled mortar with liquid nitrogen, and then 2 mL of pre-cooled cell extract buffer (20 mM potassium phosphate buffer: 0.425% KH_2_PO_4_, 0.0292% NaCl, 0.0952% MgCl_2_, 0.0154% DTT, pH 6.5) was added to mycelia [23]. Crude enzyme extract was obtained by retaining the supernatant formed after centrifugation at 4 °C and 10,000 rpm. Following previous methods [24], the activity of α-phosphoglucomutase (PGM), phosphoglucose isomerase (PGI), and phosphomannose isomerase (PMI) was determined in a total volume of 250 μL and reaction temperature of 30 °C. Enzyme activity was defined as: one unit of enzyme activity corresponded to 1 Um of NADPH conversion per minute in the reaction system. The formation of NADPH is suggested by alteration in absorbance at 340 nm[25]. Protein contents were measured by Bradford method [26].

### Scanning electron microscopy (SEM) analysis

For morphological observations of mycelial structures, ABSC mycelium was placed in 250-mL flasks and equal amounts (100 mg) of LRM anthocyanins and proanthocyanidins (99% purity, Beijing *Solarbio* Science & Technology Co., Ltd) and petunia anthocyanins (5 mg) (95% purity, SHANGHAI ZZBIO CO., LTD.) were added to each flask, and incubated for 4 days. At the end of fermentation, cells were collected by centrifugation, washed three times with sterile phosphate-buffered saline (pH 7.2, 0.8% NaCl, 0.02% KCl, 0.17% Na_2_HPO_4_, 0.8% KH_2_PO_4_) and fixed with 2.5% (v/v) glutaraldehyde. The samples were then treated progressively by 20, 30, 50, and 70% ethanol for 5 min [27] and lyophilized. Samples were then sent to the State Key Laboratory of Three Rivers Sources of the Qinghai University, China, for scanning electron microscopy (SEM JSM-7900S JEOL) analysis (5.0 kV, 10.5 mm, Secondary electrons mode).

### RNA-Seq analysis

Total RNA was extracted using Total RNA Miniprep Kit (Axygen, Hangzhou, China) following the manufacturer’s instructions. RNA quality was performed in an Agilent 2100 Bioanalyzer (Agilent Technologies Inc.). RNA-Seq libraries were prepared from cDNA generated using iScript™ cDNA synthesis kit (Bio-Rad, Hercules, CA, USA) at Sangon Biotech (Shanghai, China) and sequenced in an Illumina Hiseq4000 (Illumina Inc., San Diego, CA, USA). ORF prediction was performed using the TRINITY method (http://trinityrnaseq.sourceforge.net/). Gene expression and differential expression levels were analyzed using RSEM (http://deweylab.bio.stat.wisc.edu/rsem/) and EDGER software (http://www.biocond.uctor.org/packages/2.12/bioc/html/edgeR.html). Gene ontology (GO) enrichment analysis was performed using the goseq R package based on a Wallenius non-central hypergeometric distribution. Functional annotation of the assembled ORFs was performed using the following databases: BLASTX with the NCBI’s non-redundant database, STRING, SWISSPROT.

### Data analysis

IBM SPSS Statistics 22.0 was used to determine statistical significance. Graphs were created in Origin 9.0 (OriginLab, Northampton, MA, USA). Lowercase letters were used to indicate *P* < 0.05 level of significance; uppercase letters were used to indicate *P* < 0.01 level of significance.

## Results

### Effect of the addition of different concentrations of LRM anthocyanin extract on ABSC fermentation

**Fig. 1:**
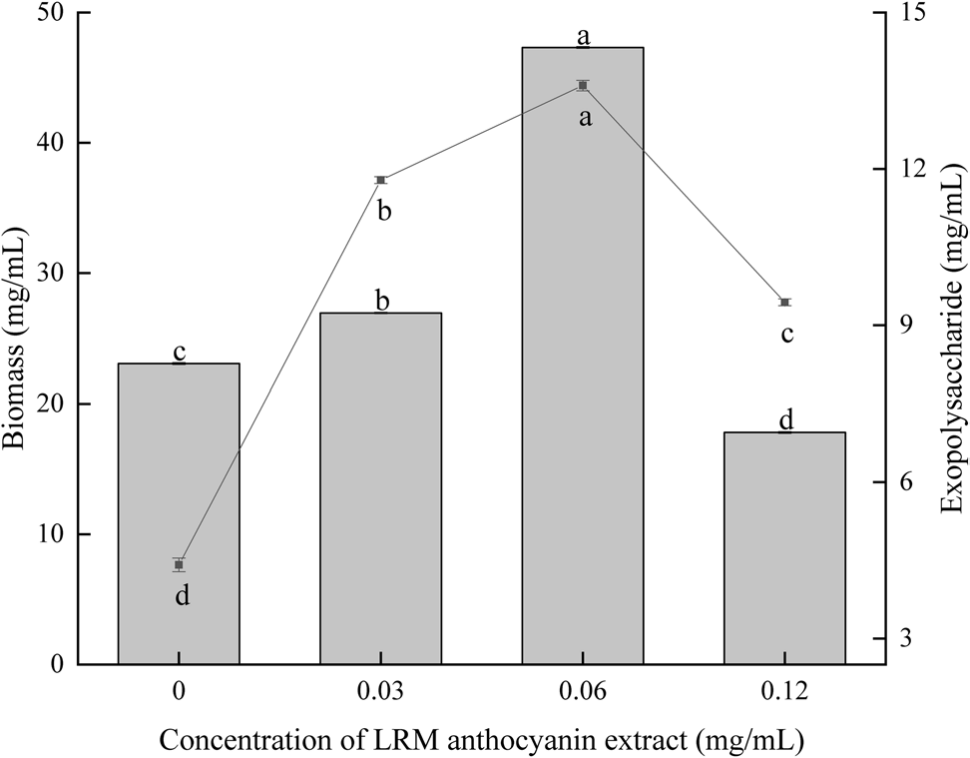
Effect of the addition of different concentrations of LRM anthocyanins on mycelial biomass and polysaccharide content during ABSC fermentation

To determine the effect of LRM anthocyanins on ABSC growth and metabolism, different concentrations (0.00, 0.03, 0.06, 0.12 mg/mL) of LRM anthocyanins extract were added to the ABSC fermentation medium, and biomass and EPS contents (mg/mL) were used as parameters to determine the optimal concentration leading to improvement in ABSC fermentation. After 5 days of fermentation, biomass and EPS content initially increased and then decreased with the addition of increasing concentrations of LMR anthocyanins (Fig. 1). Specifically, 0.06 mg/mL of anthocyanins significantly increased biomass and EPS content (*P* < 0.05). Compared with the control group (fermentation medium without the addition of anthocyanins), biomass increased by 1.05-fold (47.32 ± 0.82 mg/mL), and EPS content showed a twofold increase (13.67 ± 0.25 mg/mL). Hence, the addition of 0.06 mg/mL anthocyanins had an effective influence on the production of biomass and EPS during ABSC fermentation. To further study the effect of anthocyanin extract and its main components, i.e., proanthocyanidin and petunia anthocyanin, on ABSC fermentation, aliquots of anthocyanin crude extract, proanthocyanidin, and petunia anthocyanin extracts (0.06 mg/mL) were, respectively, added to the ABSC fermentation medium, and the effect on biomass and EPS content was investigated.

### Effect of the addition of different types of anthocyanins on ABSC biomass and EPS synthesis

**Fig. 2:**
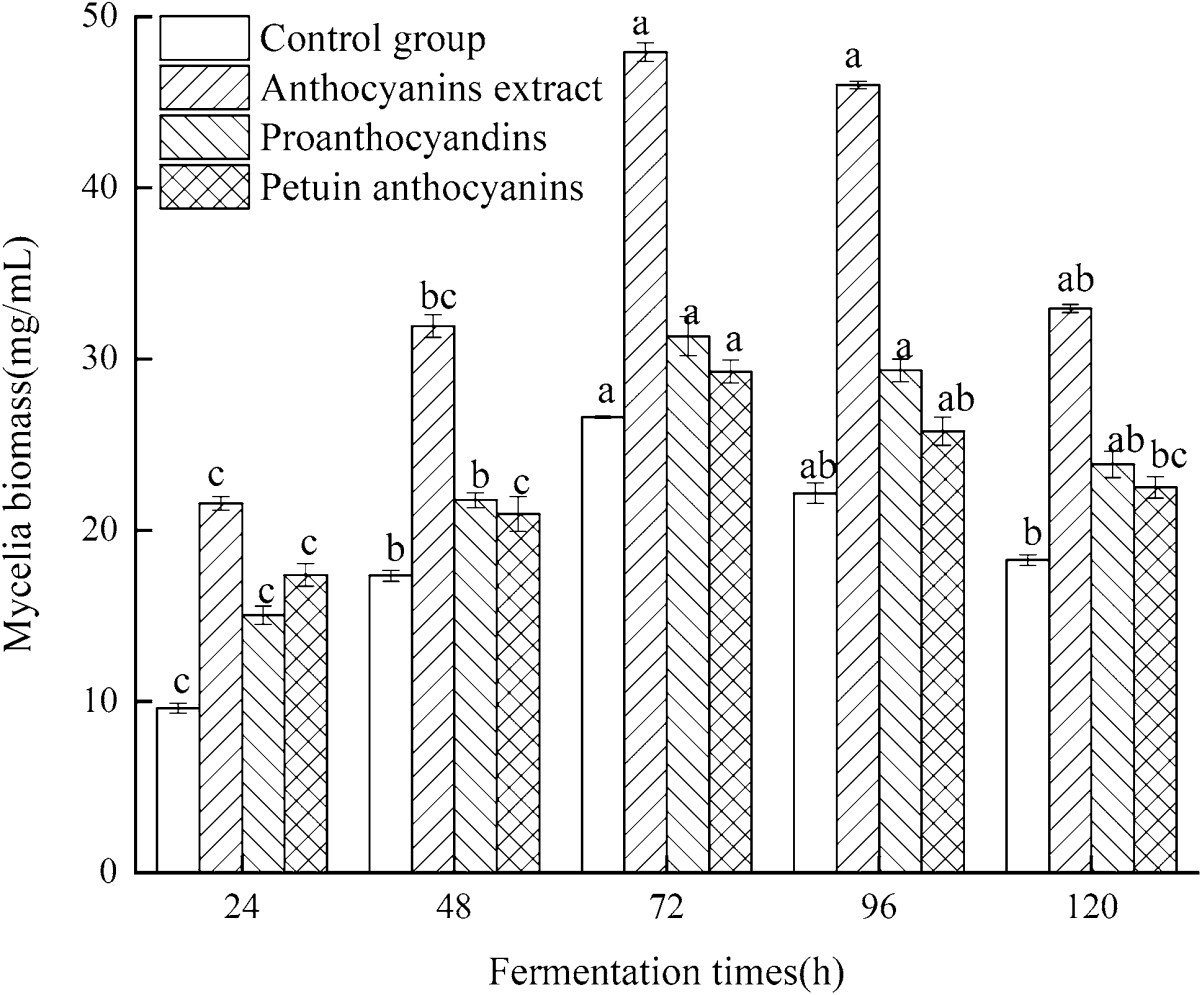
Effect of the addition of different anthocyanins on mycelium biomass accumulation during ABSC fermentation

**Fig. 3:**
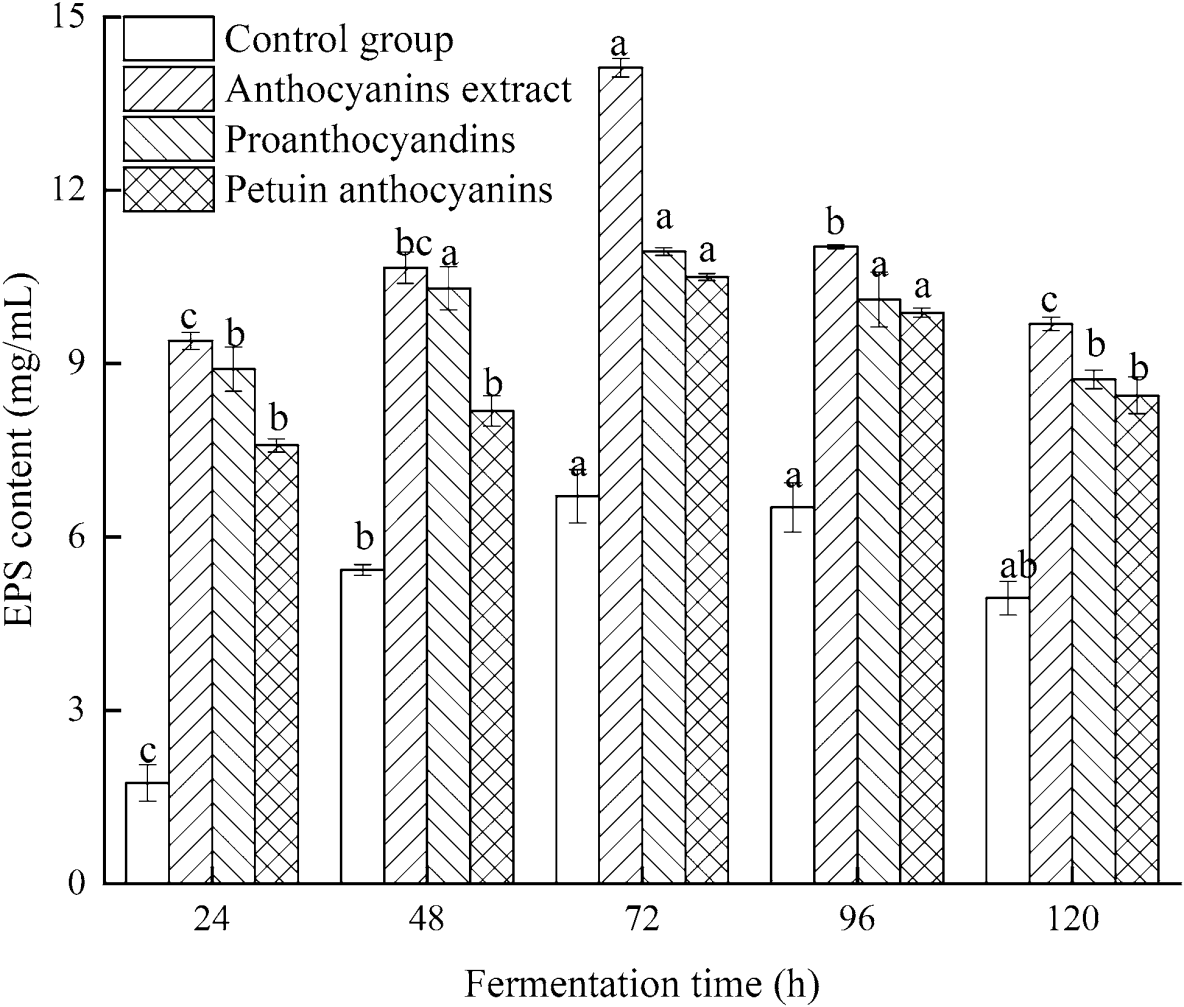
Effect of the addition of different anthocyanins extracts on exopolysaccharide (EPS) production by ABSC during fermentation

ABSC fermentation was carried out in a liquid medium containing different fractions obtained from LMR anthocyanin crude extract (0.06 mg/mL) to determine which fraction was most effective in promoting ABSC fermentation. Samples were obtained every 24 h for the determination of biomass and EPS contents and ABSC growth status. Biomass in all three experimental groups was significantly higher compared with the control (*P* < 0.05) (Fig. 3). Among added extracts, LMR anthocyanin had the greatest effect on promoting effective biomass accumulation (*P* < 0.05) (Fig. 2), followed by proanthocyanidins and petunia anthocyanin. ABSC grew well for 72 h, after which mycelia entered the decay phase, accompanied by increasing viscosity of fermentation broth and mycelial autolysis with gradual decrease in biomass. Therefore, the addition of anthocyanins to the ABSC fermentation medium promotes the accumulation of biomass produced by ABSC, and the degree of biomass accumulation is likely determined by the nature of the anthocyanin molecule added to the fermentation medium.

EPS content stimulated by the addition of anthocyanin extracts in the ABSC fermentation medium was higher compared with the control (*P* < 0.05), and peaked after 72 h of fermentation. As shown in Fig. 3, in the early stage of fermentation (24–72 h), exogenous addition of anthocyanins promoted significantly EPS accumulation. In the late stages of fermentation (96–120 h), with mycelia decay and catabolism prevailing over anabolism, a decrease in EPS accumulation was observed. Additionally, proanthocyanidins were the most effective compound to induce EPS synthesis, followed by petunia anthocyanin.

Hence, the addition of different types of anthocyanins into the ABSC fermentation medium led to remarkable changes in mycelial biomass and EPS production, as well as in mycelial growth. At 72 h of fermentation, biomass and EPS production peaked, resulting in mycelial biomass and EPS yield 3.27 and 2.11 higher, respectively, compared to the control group. Therefore, anthocyanins extracted from LRM can promote ABSC biomass accumulation and EPS production. This promoting effect is presumably related to the nature of the active component (procyanidins or petunia anthocyanin) and might be caused by a synergistic effect among the bioactive components.

### Effect of different types of anthocyanins on ABSC hyphal morphology

**Fig. 4:**
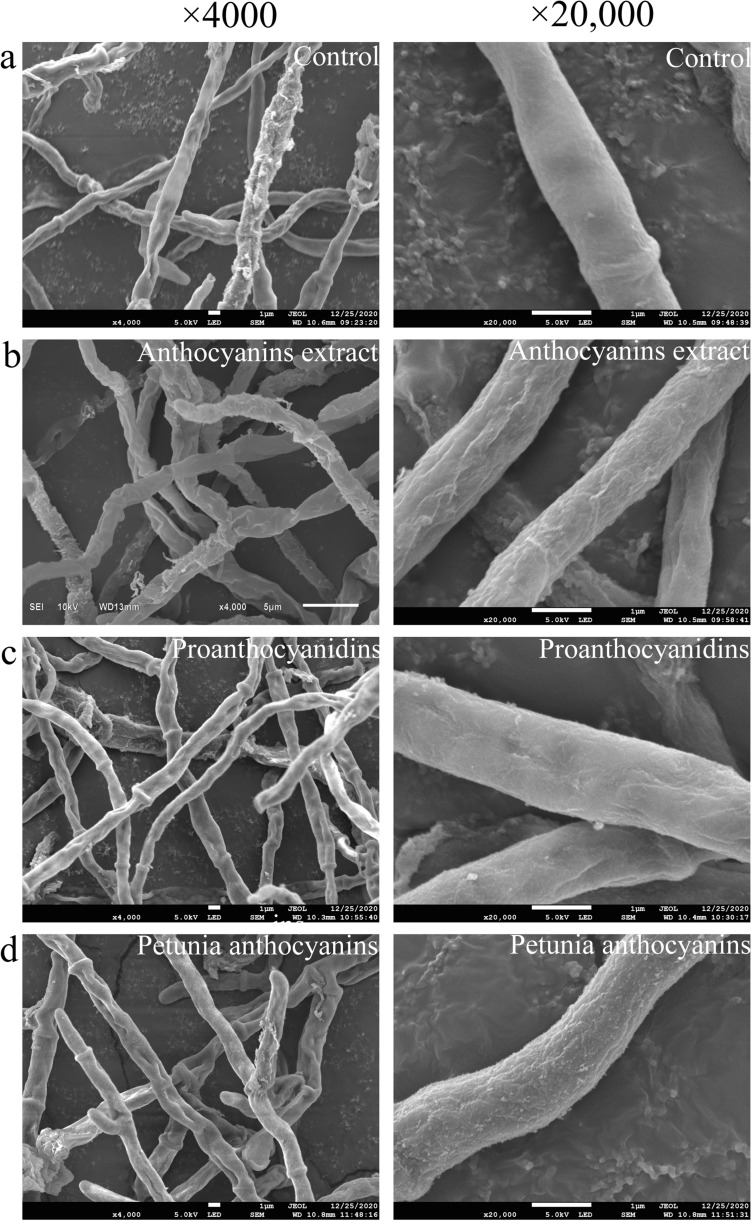
Effects of different anthocyanins on ABSC morphology during fermentation. **a** Control group, **b** anthocyanins extract, **c** proanthocyanidins, **d** petunia anthocyanins

Mycelium growth is closely related to hyphae morphology and structure. To investigate the effect of the addition of the LRM anthocyanin extract and its major components (procyanidins and petunia anthocyanins) on ABSC, fermentation was conducted for 5 days in a medium containing different anthocyanins. Mycelial morphology investigated by SEM revealed a different degree of change in ABSC hyphae structures after the addition of LRM anthocyanin extract and its major components to the fermentation medium. Mycelial density after stimulation by LRM anthocyanins and major components was altered, with mycelium showing more branches under stimulation by LRM anthocyanins. The surface of the mycelium in the control group was smooth and intact after 72 h of fermentation (Fig. 4a). In contrast, ABSC mycelium in medium containing LRM anthocyanins showed evident folds and slight indentations (Fig. 4b). Therefore, we speculated that the addition of LRM anthocyanins led to an increase in cell membrane fluidity and alterations in permeability, which was conducive to greater exchange of nutrients and elimination of metabolites, thus accelerating ABSC growth and synthesis of polysaccharides.

### Effect of LRM anthocyanins on the activity of enzymes involved in EPS synthesis

**Fig. 5:**
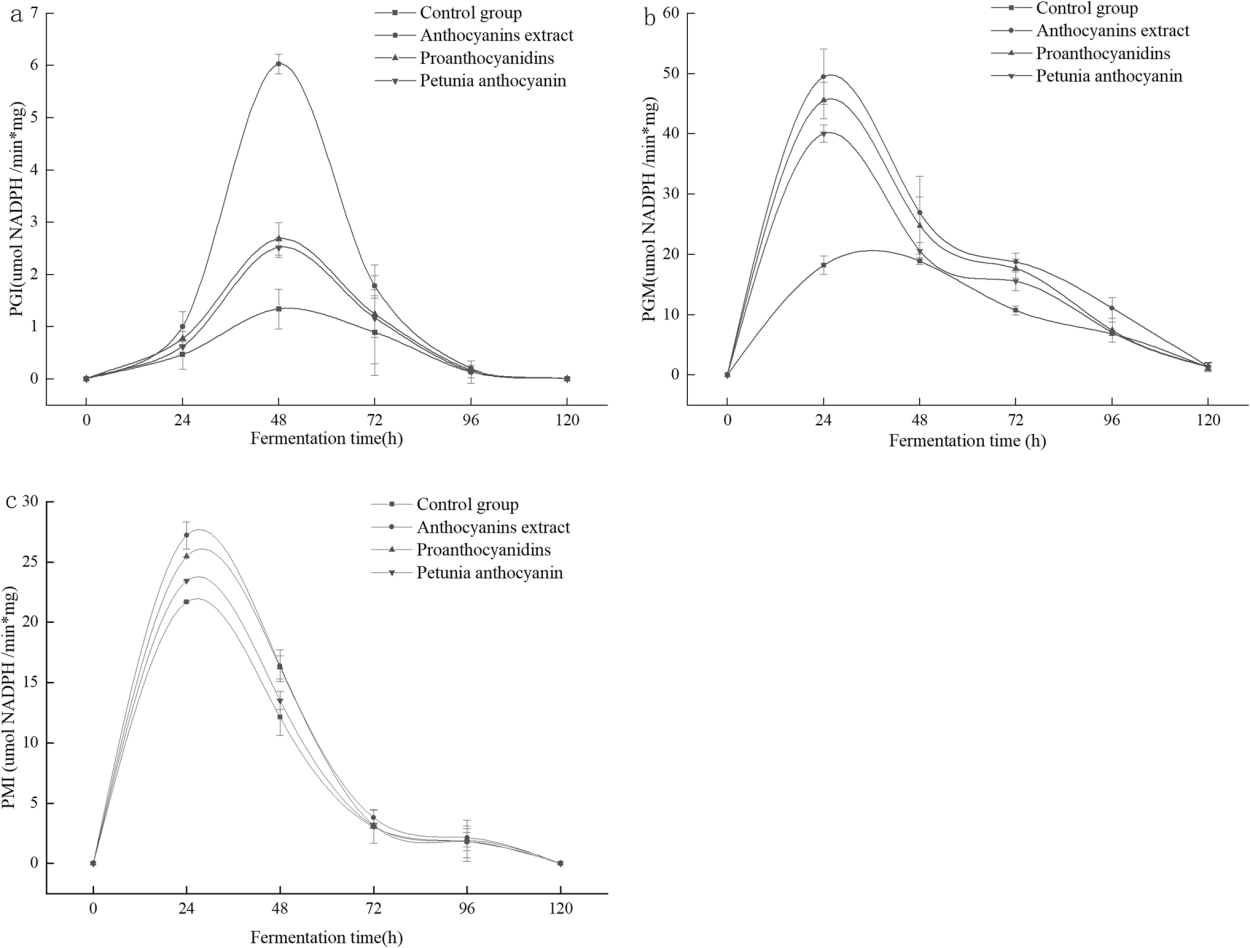
Effect of LMR anthocyanin crude extract and its main derivative fractions on the activity of PGI, PGM, and PMI involved in the synthesis of EPS by ABSC during fermentation

PGM and PGI are enzymes known to be involved in determining the composition of EPS monosaccharides in *Ganoderma lucidum* [24]. Therefore, the effect of LRM anthocyanins extract on the activity of these enzymes was investigated. PGI activity in ABSC is shown in Fig. 5. PGI activity after the addition of LRM anthocyanins increased initially and then decreased, reaching a peak at 48 h. PGI activity in the medium containing LRM anthocyanins was significantly different from that in the control group (*P* < 0.05), with an increase in PGI conversion rate by 349.9%. Interestingly, PGI activity in the medium containing proanthocyanidin or petunia anthocyanin extracts was 100% and 88% compared with the control group, respectively, which indicates a less-pronounced effect of such fractions compared to the LRM anthocyanin crude extract. Moreover, PGM activity in medium with LRM anthocyanin crude extract, proanthocyanidin, and petunia anthocyanin extracts group were 171.6, 150.0, and 120.0% (*P* < 0.05), respectively, compared with the control group.

Moreover, PMI activity during ABSC fermentation in medium with added LRM anthocyanin crude extract, proanthocyanidin, and petunia anthocyanin extracts was 20.3%, 14.8%, and 7.5% higher than in the control group (*P* < 0.05), respectively. Therefore, it can be inferred that the addition of LMR anthocyanin crude extract, and to a less-pronounced degree of proanthocyanidins and petunia anthocyanin extracts, had a remarkable effect in enhancing PGI, PGM, and PMI activity in ABSC during fermentation.

Collectively, LMR anthocyanin crude extract has been shown to increase the activity of key enzymes involved in the synthesis of EPS, thus positively affecting EPS yield. Moreover, LRM anthocyanins showed a more pronounced effect than proanthocyanidins and petunia anthocyanins in promoting the activity of a key enzyme involved in EPS synthesis. Overall, LRM anthocyanins contributed to promoting growth and EPS accumulation during liquid fermentation of ABSC, and this strategy can be applied to accelerate the fermentation process.

### Transcriptomic and functional analysis of ABSC by RNA-Seq

**Fig. 6:**
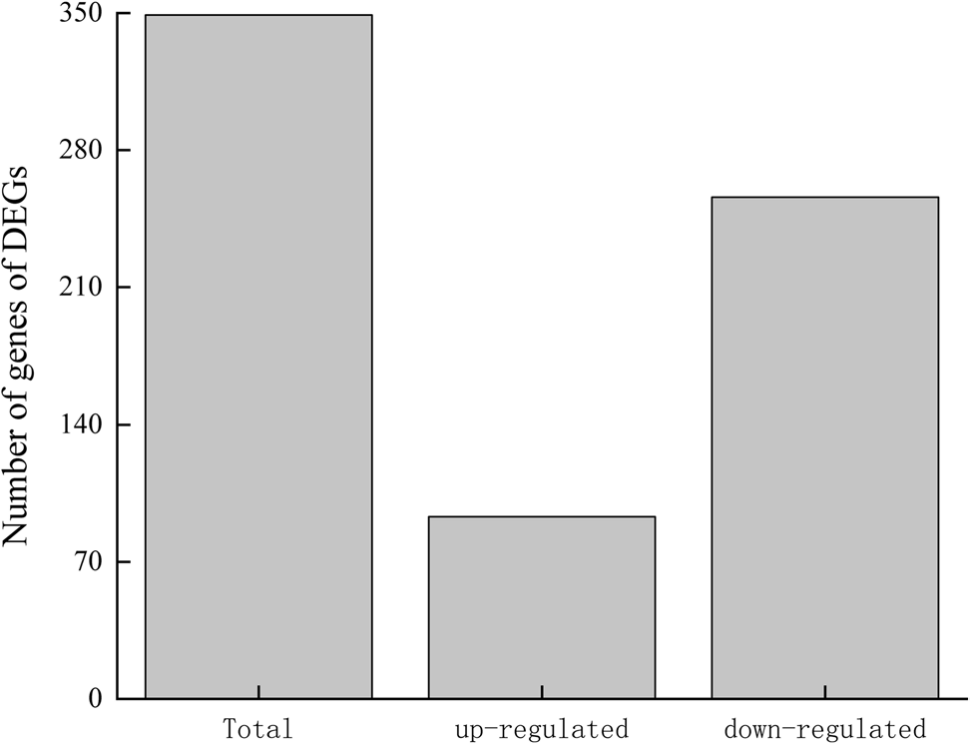
The number of differentially expressed genes (DEGs; log_2_ fold change) of ABSC grown in the absence or presence of LRM anthocyanins

**Fig. 7:**
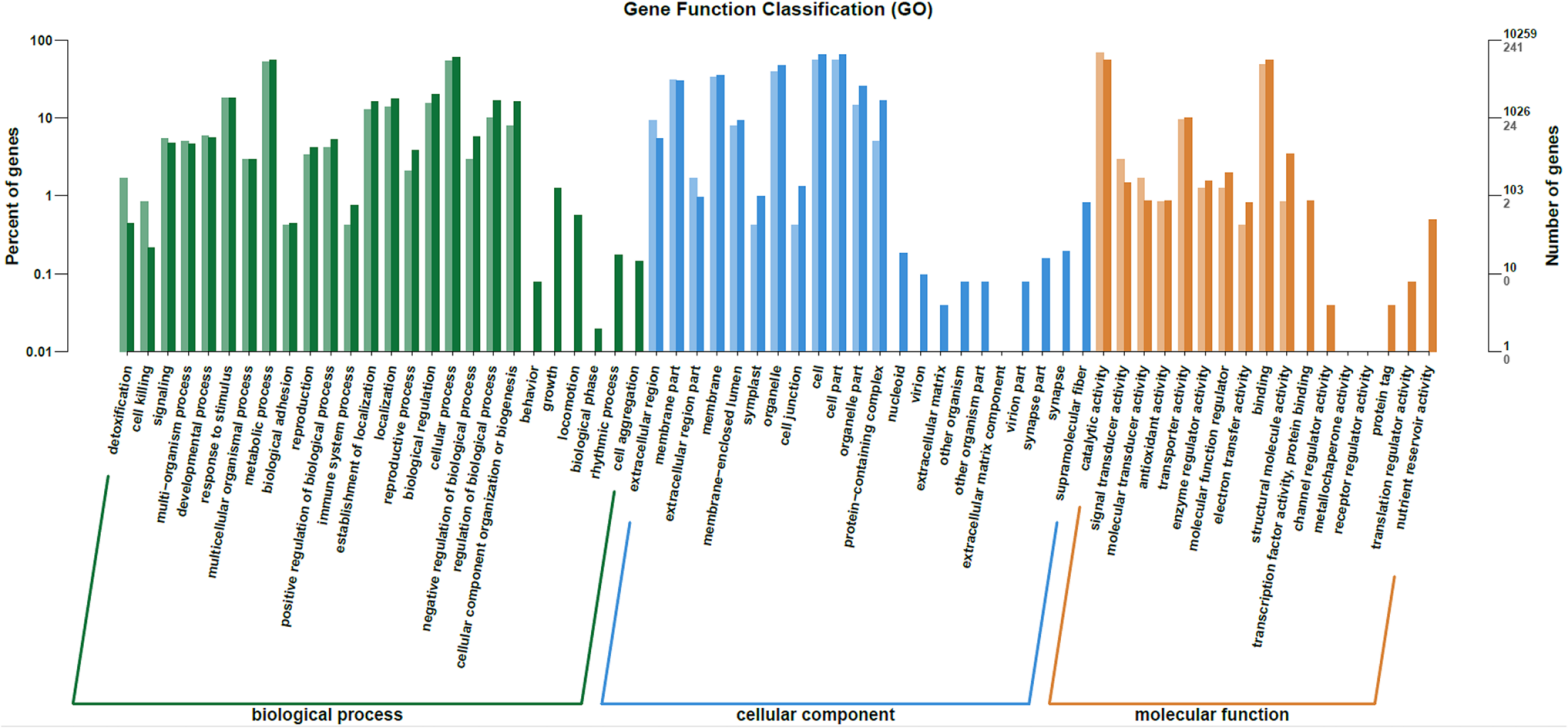
Gene ontology enrichment analysis of differentially expressed genes of ABSC

To further explore the mechanism underlying the LRM anthocyanin-mediated effect on EPS synthesis in ABSC, RNA-Seq analysis was conducted to evaluate the transcriptomic response of ABSC in medium containing LRM anthocyanins. Transcriptomic data revealed a total of 56,115 transcripts; the average transcript size was 3450.28 bp, average GC content was 53.73%, and Q30 score was between 96.14 and 96.16% (Table 1). A total of 349 genes were differentially expressed, among which 93 genes were up-regulated and 256 genes were down-regulated (log_2_ Fold Change ≥ 2, False discovery rate (FDR) < 0.05) (Fig. 6). Gene ontology (GO) enrichment analysis was used to assign and quantify transcripts according to their presumed function in the cell (Fig. 7). GO analysis indicated 22 subgroups in cellular components, 17 subgroups in molecular functions, and 26 subgroups in biological processes. Differentially expressed genes (DEGs) in the cellular components category mainly included cell, cell part, organelle, and cell membrane. Within the molecular function category, the highest number of transcripts of DEGs were assigned to binding and catalytic activity. In addition, a high number of transcripts were included in biological processes, cell processes, metabolic processes, and response to stimulus categories.

**Table 1:** Summary of raw RNA-Seq data of ABSC grown in medium with and without LRM anthocyanins

Samples	Number of reads	Number of bases covered	GC content (%)	%Q30
ABSC grown in control fermentation medium	58,229,850	8,148,180,728	53.58	96.14
ABSC grown in fermentation medium containing anthocyanin extract	54,491,054	7,705,162,685	53.73	96.16

**Fig. 8:**
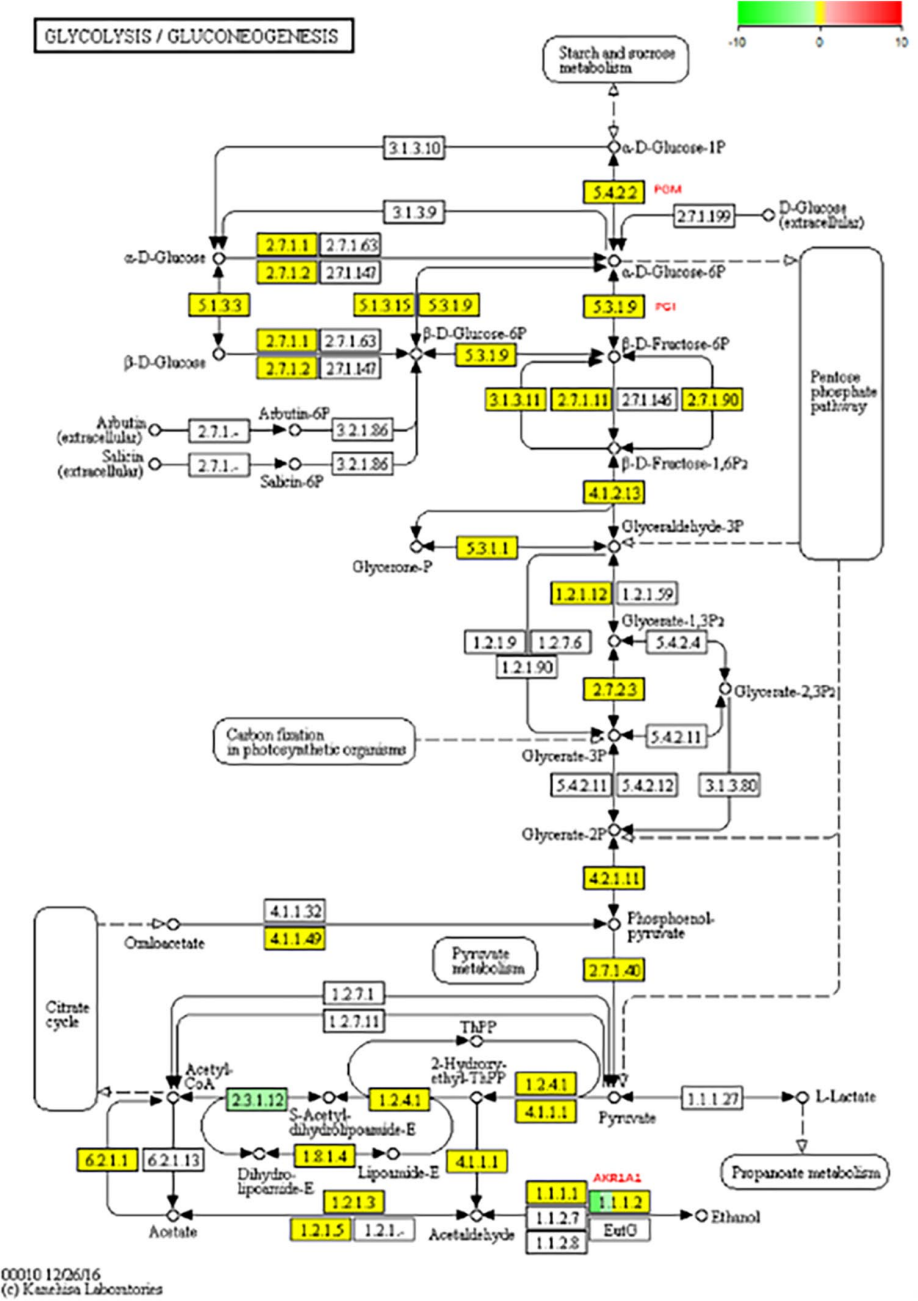
Differentially expressed genes (yellow boxes) and down-regulated genes (green boxes) of ABSC involved in the gluconeogenesis/glycolytic metabolic pathway during growth in medium with or without LRM anthocyanidin extract

Levels of PGI and PGM in ABSC grown in the presence of LRM anthocyanins extract varied compared with the control group (Fig. 5), but no significant differences in the expression of genes involved in key metabolic pathways were observed (Fig. 8). Findings discussed earlier indicated that activity of PGI and PGM was greater in treated groups than in the control group. Therefore, LRM anthocyanins extracts alter the activity of PGI and PGM at the protein level, but do not induce changes at the transcription sites of polysaccharide-related genes.

## Discussion

Polysaccharides from edible and medicinal fungi have a variety of biological functions, such as anti-tumor, antiviral, antioxidant, and immunomodulatory properties [4–6]. In recent years, the method for obtaining polysaccharides produced by such fungi through submerged fermentation has become more sophisticated [28, 29]. Optimization of culture condition and medium composition has shown to be essential for promoting adequate mycelial growth and production of polysaccharides. It has been reported that some exogenous additives or regulatory factors increase the mycelial growth of edible fungi and lead to an accumulation of active fungal metabolites and might also induce changes in metabolites structure hence affecting their biological activity [8, 30]. Xu et al. [31] reported that the addition of *Gastrodia elata* extract during fermentation of the fungus *Grifola frondosa* promoted its growth and increased mycelial biomass by 1.06-fold. Lee et al. [32] found that the addition of ascorbic acid at low concentration (0.1 mg/mL) to the liquid fermentation medium of *Hericium erinaceus* could effectively promote hyphae growth and led to a significant increasing in the yield of polysaccharide. Moreover, Yang et al. [33] used liquid fermentation medium with 2% *Coix Lacryma-Jobi* oil during growth of *Ganoderma lucidum*, which led to an increase in mycelial biomass (3.34-fold) and higher recovery of triterpenes, EPS, and IPS (2.76-, 2.2-, and 2.23-fold, respectively). Collectively, the results showed that the LRM anthocyanins crude extract acted as a positive regulatory factor, which promoted growth of and increased metabolite production by ABSC. Moreover, the main fractions present in the crude extract (proanthocyanidins and petunia anthocyanins) seemingly played an important role in the observed phenomenon. At optimal supplementation level (0.06 mg/mL), the biomass of ABSC and EPS yield was 47.32 mg/mL and 13.67 mg/mL, which were higher by 1.05- and 2.08-fold, respectively, compared to the control group (Fig. 1). However, the higher anthocyanins extract of LRM had an inhibitory effect on the growth of ABSC. This is similar to the study of Xiang et al. [34], indicating that the alcohol extract of LRM can inhibit a variety of microorganisms such as *Escherichia coli*, *Staphylococcus aureus*, and *Salmonella*, the inhibitory ability of bacteria to fungi gradually decreased. In addition, anthocyanins of LRM may also contain active substances such as betaine and tannins [35], which denaturant cell membrane proteins and thus inhibit microbial growth [36].

LRM-derived anthocyanins are rich in proanthocyanidins and other types of anthocyanins, among which petunia anthocyanins are the main representatives [11]. To evaluate the effect of LRM anthocyanins on ABSC during fermentation, LRM anthocyanin crude extract and its main fractions (proanthocyanidins and petunias anthocyanins) were added to the fermentation medium at different concentrations. The effect was preliminarily analyzed by assessing mycelial biomass accumulation and EPS yield. The present findings revealed that anthocyanin crude extract and its main component fractions had different effects on ABSC. Interestingly, LRM anthocyanin crude extract showed the best ABSC growth-promoting effect compared with the use of single anthocyanins. At 72 h of fermentation, biomass and EPS production peaked, which was 3.27- and 2.11-fold higher, respectively, than that of the control group. Previous studies have found that plant oils are recommendable stimulants rather than individual fatty acids to *Cordyceps militaris* fermentation and metabolism [37]. Therefore, it can be assumed that during fermentation, anthocyanin extract had a composite effect on ABSC, which can be likely attributed to the synergistic effect among the various bioactive substances present in the crude extract. Moreover, fungi mycelial morphology is known to be related to the production of secondary metabolites [38]. The present findings showed that the three exogenous additives evaluated in this study had different effects on mycelium morphology of ABSC, which may affect biomass and EPS production. Similarly, Lee et al. [39] evaluated the addition of surfactant to *Trichoderma harzianum* fermentation culture medium and observed that changes in fungal morphology from aggregation to dispersion affected the accumulation of cellulase.

The proposed stimulatory effect linked to the exogenous addition of oil or fatty acids to liquid fermentation of edible macrofungi can be attributed to changes in membrane composition and increased membrane permeability, or by directly affecting the synthesis rate of enzymes involved in stimulating metabolism [33, 37, 40]. PGM and PGI are key enzymes in the synthesis of *G*. *lucidum* polysaccharide [24]. Moreover, it has been previously described that the stimulating effect of *Coix lacryma-Jobi* oil on *Ganoderma lucidum* polysaccharide production might be related to the synthesis of PGM and PGI [33]. In addition, coixenolide has been shown to increase the synthesis of *G. lucidum* EPS. Herein, LRM anthocyanin extract and its other related components affected EPS production in ABSC through PGI, PGM, and PMI to varying degrees, with LRM anthocyanin crude extract showing the best-promoting effect. Similarly, Zhou et al. [30] described that the stimulatory mechanism of coixenolide on *G. lucidum* polysaccharides would directly affect the synthesis of PGM and PGI. In the present study, the transcriptomic analysis showed the anthocyanins extract of LRM could significantly affect carbohydrate metabolism and change the direction of carbon flux. The expression genes of PGI and PGM were different partly in the process of gene transcription, but the expression was not significant (Fig. 8). However, the results showed that the enzyme activities of PGI and PGM improved to different extend under the influence of anthocyanins, which indicated anthocyanins or its decomposing compounds could change the content of ROS [41] or combine with enzyme activity centers [42], thereby changing the activities of the related enzymes. Collectively, the present findings revealed that the use of anthocyanin extract from LRM as inducers significantly altered liquid fermentation of ABSC. Among anthocyanins recovered from LRM, procyanidins and petunia anthocyanin contributed greatly to the growth- and EPS-promoting effect. Further studies are needed to evaluate the different properties of the LRM anthocyanin extract on fungi, as well as to elucidate which compounds have growth- and EPS-promoting effects and whether the addition of such compounds during fermentation of ABSC affects their structure and activity.
