# Correction to: Anthocyanin extract from *Lycium ruthenicum* enhanced production of biomass and polysaccharides during submerged fermentation of *Agaricus bitorquis* (Quél.) Sacc. Chaidam

**DOI:** 10.1007/s00449-022-02724-w

**Published:** 2022-03-26

**Authors:** Shan Wu, Hong‑Yun Lu, Qi‑He Chen, Hui‑Chun Xie, Ying‑Chun Jiao

**Affiliations:** 1 College of Agriculture and Animal Husbandry, Qinghai University, No.251 Ningda Road, Xining, 810016 Qinghai China; 2 Department of Food Science and Nutrition, Zhejiang University, 154 Yugu Road, Hangzhou, 310058 Zhejiang China; 3 Qinghai Provincial Key Laboratory of Medicinal Plant and Animal Resources of Qinghai‑Tibet Plateau, School of Life Sciences, Qinghai Normal University, Xining, 810008 Qinghai China

## Correction to: Bioprocess and Biosystems Engineering (2021) 44:2303–2313 10.1007/s00449-021-02605-8

The Acknowledgements section should be modified as.

This work was financially supported by the Natural Science Foundation of China (31,960,471) and the Science and Technology Project of Qinghai (No. 2020ZJY40).

